# 934 Burn-Injured Females More Prone to Chronic Shame Than Male Counterparts and Family Support Matters

**DOI:** 10.1093/jbcr/iraf019.465

**Published:** 2025-04-01

**Authors:** Ruth Rimmer, Curt Bay, Emile Kalil, Daniel Chacon, Erika Mendoza, Tess Robaina, Kevin Foster

**Affiliations:** Diane & Bruce Halle Arizona Burn Center; A. T. Still University; University of Maryland School of Medicine; Alisa Ann Ruch Burn Foundation; Arizona State University; Arizona Burn Foundation; Diane & Bruce Halle Arizona Burn Center

## Abstract

**Introduction:**

Several psychological disorders, including PTSD, depression and anxiety, have been noted to occur in burn-injured persons commonly. Shame and guilt, distinct constructs, can also follow burn injury. Shame after trauma, a maladaptive response, has been linked to negative outcomes, including suicidality, substance abuse, and identity issues.

**Methods:**

Burn-injured individuals were invited to complete the Guilt Voluntarily and Shame Proneness Scale (GASP). It measures individual differences in the tendency to experience guilt and shame across a range of personal transgressions. The GASP contains four four-item subscales: Guilt-Negative-Behavior-Evaluation (Guilt-NBE), Guilt-Repair, Shame-Negative-Self-Evaluation (Shame-NSE), and Shame-Withdraw.

**Results:**

Participants included burn survivors (n=116), with a mean age of 16.3 years, SD (±3.96), female (n=62), male (n=52), Caucasian (25%), Hispanic (48%), and African American (18%), the average age at burn was 6 years, with 52% reporting visible burn scars. Females reported significantly higher scores on the Shame-NSE scale (p=0.020). Those describing “just right” & moderate family support (63%) had significantly lower shame scores (p=0.008). There were no differences based on the visibility of scars or ethnicity.

**Conclusions:**

Burn-injured females report experiencing high levels of shame after being burned. Multiple studies have revealed a significant relationship between shame and negative internal traits. Individuals with high levels of internalized shame are more likely to suffer from low self-esteem and negative self-worth. Unresolved shame is linked to serious psychopathology, so intervention to address this problem for burn-injured females is recommended. Family support should also be encouraged as a preventative measure.

**Applicability of Research to Practice:**

Because shame proneness is associated with negative self-evaluation and feelings of incompetency and inferiority, burn care professionals should consider screening female survivors for post-traumatic shame. When high levels of shame are present, individuals should be referred for further psychological evaluation. World Burn Congress, local burn support groups, and retreats should be recommended and considered, offering interventions to help deal with and resolve shame.

**Funding for the Study:**

N/A

